# Association of smoking and cardiovascular disease with disease progression in COVID-19: a systematic review and meta-analysis

**DOI:** 10.1017/S0950268821001138

**Published:** 2021-05-12

**Authors:** Shiwei Kang, Xiaowei Gong, Yadong Yuan

**Affiliations:** Department of Respiratory and Critical Care Medicine, The Second Hospital of Hebei Medical University, Shijiazhuang, China

**Keywords:** ACE2, cardiovascular disease, COVID-19, meta-analysis, smoking

## Abstract

The aim of this study was to systematically assess the association between smoking and cardiovascular disease (CVD) and disease progression among novel coronavirus pneumonia (coronavirus disease 2019 (COVID-19)) cases. PubMed database and Cochrane Library database were searched by computer to seek the epidemiological data of COVID-19 cases and literatures regarding CVDs from 1 Jan to 6 October 2020. Two researchers independently conducted literature screening, data collection and the assessment of the risk of bias of the studies included. RevMan 5.2 software was employed for meta-analysis. Funnel plot was adopted to assess the publication bias. On the whole, 21 studies comprising 7041 COVID-19 cases were included. As revealed from the meta-analysis, 14.0% (984/7027) of cases had a history of smoking, and 9.7% (675/6931) were subject to underlying CVDs. Cases with a history of smoking achieved a higher rate of COVID-19 disease progression as opposed to those having not smoked (OR 1.53, 95% CI 1.29–1.81, *P* < 0.00001), while no significant association could be found between smoking status and COVID-19 disease progression (OR 1.23, 95% CI 0.93–1.63, *P* = 0.15). Besides, smoking history elevated the mortality rate by 1.91-fold (OR 1.91, 95% CI 1.35–2.69, *P* = 0.0002). Moreover, underlying CVD elevated the incidence of severe disease by 2.87-fold (OR 2.87, 95% CI 2.29–3.61, *P* < 0.00001) and mortality by 3.05-fold (OR 3.05, 95% CI 1.82–5.11, *P* < 0.0001) in COVID-19 cases. As demonstrated from the current evidence, smoking displays a strong association with COVID-19 disease progression and mortality, and intensive tobacco control is imperative. Moreover, cases with CVD show a significantly elevated risk of disease progression and death when subject to COVID-19. However, the association between COVID-19 and CVD, and the potential effect exerted by smoking in the development of the two still require further verifications by larger and higher quality studies.

## Introduction

An outbreak of novel coronavirus pneumonia (coronavirus disease 2019 (COVID-19)) occurred in Wuhan, China in December 2019, which presented an outbreak situation and has now caused a global epidemic. The disease is caused by a novel coronavirus (severe acute respiratory syndrome-coronavirus 2 (SARS-CoV2)), which belongs to the novel beta genus coronavirus and is mainly characterised by strong transmission and high pathogenicity [1]. As of 27 March 2021, the World Health Organization (WHO) has received a total of more than 125 million confirmed cases, including over 2.7 million deaths. As indicated from the epidemic report, the epidemic is spreading and accelerating continuously, and all mankind faces great challenges [2].

For a long time, smoking has been generally accepted to display a close relationship to the poor prognosis of lung diseases. There is substantial evidence that smoking negatively affects lung health [3]. In addition, ex vivo and in vivo studies suggested that smoking is capable of elevating the risk of respiratory tract infection by causing chronic lung inflammation and destructive immune response [4]. Moreover, it elevates the risk of heart disease, cancer and other diseases in smokers and surrounding people. However, the association between smoking and COVID-19 disease remains controversial. Lippi *et al*. [5] did not identify any significant association between COVID-19 disease severity and disease development and smoking by conducting a meta-analysis on five studies; while the results of study conducted by Karanasos *et al*. [6] suggested that smoking may increase disease severity and mortality in hospitalised COVID-19 cases.

In addition, considerable clinical studies have shown that severe COVID-19 cases are often associated with a variety of underlying diseases, especially chronic underlying diseases represented by cardiovascular diseases (CVDs) (e.g. coronary heart disease). Recent studies have shown that COVID-19 cases subject to underlying CVDs have a worse prognosis than ordinary cases, and some cases experience myocardial injury associated with viral infection [7]. The results of Wang *et al*. [8] showed that more than 7% of COVID-19 cases had myocardial injury, accounting for 22% of critically ill cases. Data suggest that CVD accounts for one-third of smoking-related deaths; even if only one cigarette is smoked daily, the incidence of coronary atherosclerotic heart disease (CAD) and stroke is significantly higher [9]. Although studies regarding the association between smoking or CVD and COVID-19 have been published, the association between smoking and CVD and the role of the two in the development of the disease in COVID-19 cases require a systematic summary.

Therefore, the present study systematically collected the relevant literature and used meta-analysis to comprehensively analyse the association between smoking and CVD and disease progression in COVID-19 cases to scientifically underpin the screening, prevention and treatment of high-risk COVID-19 cases.

## Materials and methods

### Literature search strategy

By complying with the PICOS principle of Cochrane Handbook of Systematic Reviews, search strategy with the goal of recall is developed, and the study results are reported and discussed in accordance with (PRISMA-P) and MOOSE specifications. Two investigators independently searched PubMed and Cochrane Library databases, supplemented by manual search, to collect and report the past smoking history and CVD-related literatures of severe, severe and dead COVID-19 cases. For the published literatures with the search date from 1 Jan to 6 October 2020, the search method combining free words and subject headings was adopted, which was regulated by considering the characteristics of different databases. Only articles in English and online publications were included in the study. Details of literature search strategy are available in Supplementary Material.

### Inclusion and exclusion criteria

*Inclusion criteria*: (1) Published relevant studies on the clinical manifestations of hospitalised COVID-19 cases comprising or containing smoking history or CVD data, and the study types are case−control study, cohort study, as well as cross-sectional study; (2) the study subjects are hospitalised COVID-19 cases diagnosed over the age of 18 years; (3) the study contents involve the smoking status, prevalence of CVD, corresponding severity of illness and disease outcome of COVID-19 cases. In this study, we defined severe COVID-19, a composite outcome, as severe COVID-19 cases (including critical cases) along with either the requirement of ICU admission, invasive ventilation, high-intensity medical care or resulting in death. The deterioration of the patient's condition to severe COVID-19 was defined as disease progression. CVD was defined as having a history or comorbidity of cardiovascular or cardiac disease in our study. Hypertension/heart failure/stroke in specific terms was excluded because these diseases often overlap, which may lead to overestimation.

*Exclusion criteria*: (1) Literature published repeatedly in the identical study; (2) non-English literatures; (3) review, short case report, conference abstract and letter; (4) literature with incomplete or missing data and data unavailable to contact authors.

### Literature screening and data extraction

Two investigators independently screened the literatures, extracted the data and cross-checked by complying with the inclusion and exclusion criteria. In case of any disagreement, it was resolved by discussion or consultation with the third investigator. The main content of data extraction: (1) basic information of the study included (e.g., the first author of the study, publication time, region and study type); (2) baseline characteristics of the study subjects (e.g. smoking status, number of smokers, population smoking rate, sample size of the case group and the control group, as well as outcome indicators); (3) vital elements of risk of bias assessment.

### Assessment of risk of bias of studies included

The Newcastle-Ottawa Literature Quality Assessment Scale (NOS) was adopted to assess the risk of bias of the studies included, and any disagreements were resolved by discussion with a third researcher. The studies included were scored from three aspects: the selection of study subjects, comparability between groups and exposure factors (overall 8 items), with a full score of 9, 0−4 as low-quality studies, as well as 5−9 as high-quality studies.

### Statistical analysis

Meta-analysis on the data was conducted with the RevMan 5.2 software package offered by the Cochrane Collaboration. Where available, adjusted effect estimates were combined and in the absence of adjustment for confounders, raw effect estimates were combined in the study. Enumeration data were expressed as effect size using odds ratio (OR), which had the expression of 95% confidence interval (CI). Under the heterogeneity test results of the studies included as *I*^2^ ⩽ 50%, the fixed-effect model was adopted for meta-analysis; under the heterogeneity test results of *I*^2^ > 50%, the random-effect model was employed for meta-analysis. Significant clinical heterogeneity was dealt with by subgroup analysis or sensitivity analysis. Publication bias was assessed by plotting funnel plots.

## Results

### Literature search results and quality assessment

**Fig. 1.**
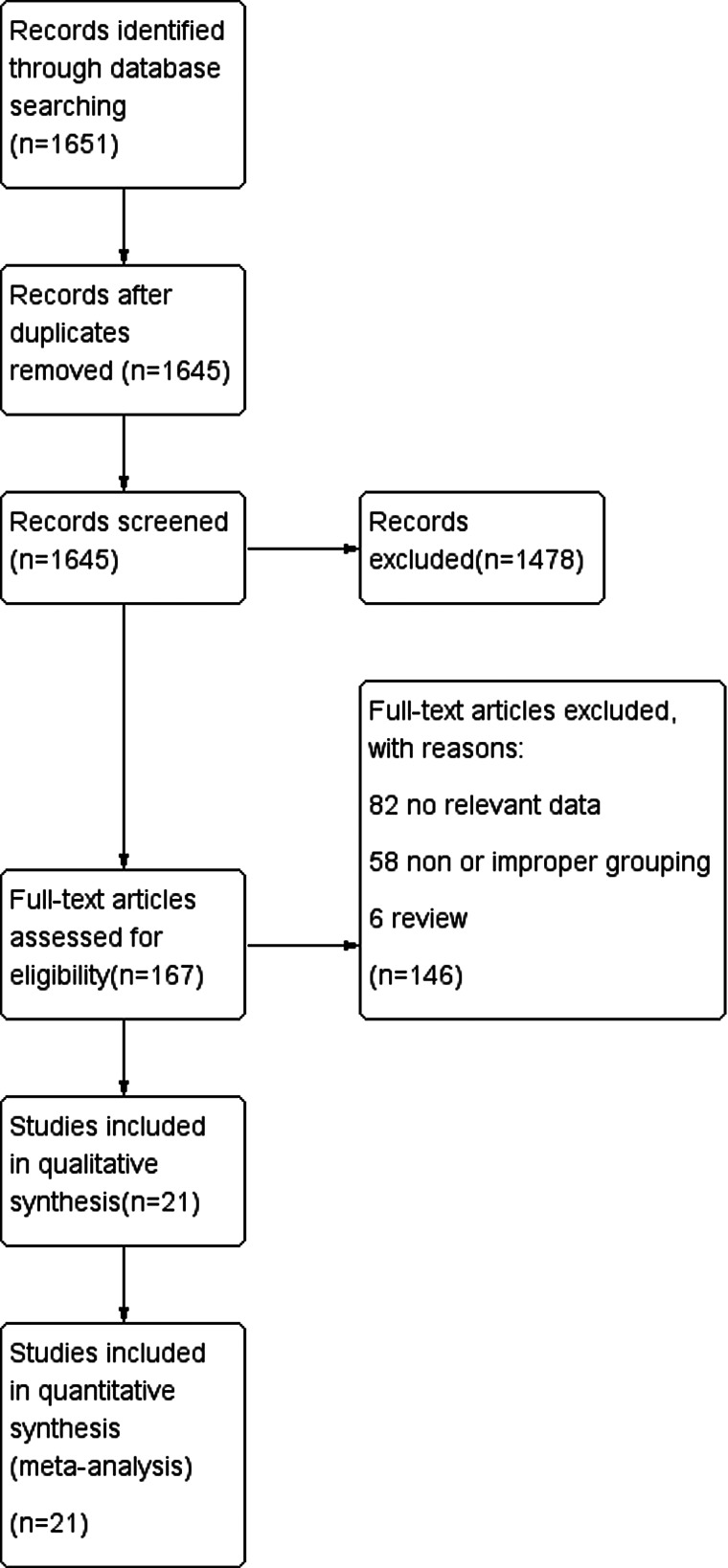
Study flow diagram.

On the whole, 1651 studies were obtained from the search database, six duplicate studies were excluded, 1478 studies were excluded after reading the titles and abstracts, 146 studies were excluded after carefully reading the full texts and screening by complying with the inclusion and exclusion criteria, and finally 21 studies [10–30] were included, all of which were retrospective cohort studies (Fig. 1). Seventeen of the studies were from China, and the remaining four were from the United States, United Kingdom, Japan and Italy. The exposure factors in this study were smoking history (including current or ex-smoking currently having quit smoking). Five studies assessed whether cases were currently smoking, six studies stratified cases by whether they were current or former smokers and the remaining 10 recorded whether cases had a history of smoking.

The result observed here was the presence of disease progression. To be specific, nine studies adopted disease severity as the outcome measure, six studies used death, two studies used disease progression, two studies used cases admitted to the ICU and the other two studies applied cases requiring upgrading of treatment regimen and requiring invasive ventilation as the outcome measures, respectively. Nineteen of the mentioned studies reported results in cases with CVD.

### Basic information of included literatures

Among the 21 retrospective cohort studies included, comprising a total of 7041 COVID-19 cases, 2010 cases (28.6%) had disease progression, 984 (14.0%) had a history of smoking and 675 (9.6%) had concomitant CVD. The NOS quality scores of the studies included were 5 and above, all of which were high-quality studies, and the basic characteristics and quality assessment are listed in Tables 1 and 2.

**Table 1.** Characteristics of patients included in the smoking analysis cohort

	Region	Type of study	Sample size	Smoking				Outcome	NOS
				Smoking status	Smokers	Severe smokers/severe cases	Non-severe smokers/Non-severe cases		
Cen *et al*. [10]	Wuhan, China	Retrospective, multi-centre observational	1007	Smoking history	88	18/287	70/720	Disease progression	7
Chen *et al*. [11]	Taizhou, Zhejiang, China	Retrospective, single-centre, observational	145	Smoking history	15	3/43	12/102	Disease severity	6
Chen *et al*. [12]	Wuhan, China	Retrospective, single-centre, observational	274	Smoking history	Current smoker 12	7/113	5/161	Death	7
					Former smoker 7	2/113	5/161		
Cocconcelli *et al*. [13]	Padova, Italy	Retrospective, single-centre, observational	102	Smoking history	Current 9	1/31	8/71	Medical care intensity	7
					Former 43	19/31	24/71		
					Non-smokers 50	9/31	41/71		
Goyal *et al*. [14]	New York, USA	Retrospective, multi-centre observational	393	Current smoking	20	6/130	14/263	Invasive ventilation	5
Guan *et al*. [15]	Nationwide, China	Retrospective, multi-centre observational	1099	Smoking history	Never smoked 927	134/172	793/913	Disease severity	6
					Former smoker 21	9/172	12/913		
					Current smoker 137	29/172	108/913		
Huang *et al*. [16]	Wuhan, China	Retrospective, single-centre, observational	41	Current smoking	3	0/13	3/28	ICU admission	7
Huang *et al*. [17]	Jiangsu, China	Retrospective, multi-centre, observational	202	Smoking history	16	2/23	14/179	Disease severity	7
Khalil *et al*. [18]	London, England	Retrospective, single-centre, observational	204	Smoking history	88	28/53	60/151	Death	7
Li *et al*. [19]	Wuhan, China	Retrospective, single-centre, observational	544	Smoking history	Never smokers 452	214/265	238/279	Disease severity	7
					Former smokers 51	33/265	18/279		
					Current smokers 41	18/265	23/279		
Ishii *et al*. [20]	Nationwide, Japan	Retrospective, multi-centre observational	345	Smoking history	117	10/23	107/322	Death	7
Shu *et al*. [21]	Wuhan, China	Retrospective, single-centre, observational	571	Smoking history	Former smoker 56	10/26	46/545	Disease progression	7
					Current smoker 80	4/26	76/545		
Wan *et al*. [22]	Chongqing, China	Retrospective, single-centre, observational	135	Current smoking	9	1/40	8/95	Disease severity	7
Wang *et al*. [23]	Fuyang, Anhui, China	Retrospective, single-centre, observational	125	Current smoking	16	7/25	9/100	Disease severity	6
Wang *et al*. [24]	Wuhan, China	Retrospective, single-centre, observational	110	Smoking history	26	9/38	17/72	Disease severity	7
Wang *et al*. [25]	Wuhan, China	Retrospective, single-centre, observational	59	Smoking history	9	9/41	0/18	Death	7
Xie *et al*. [26]	Nationwide, China	Retrospective, multi-centre observational	733	Smoking history	45	33/394	12/339	Death	7
Yang *et al*. [27]	Yichang, China	Retrospective, single-centre, observational	200	Smoking history	9	1/29	8/171	ICU admission	7
Zhan *et al*. [28]	Wuhan, China	Retrospective, single-centre, observational	405	Smoking history	46	25/148	21/257	Disease severity	7
Zhang *et al*. [29]	Wuhan, China	Retrospective, single-centre, observational	140	Smoking history	Past smokers 7	4/58	3/82	Disease severity	7
					Current smokers 2	2/58	0		
Zhou *et al*. [30]	Wuhan, China	Retrospective, multi-centre observational	191	Current smoking	11	5/54	6/137	Death	7

**Table 2.** Characteristics of patients included in the CVDs analysis cohort

	Region	Type of study	Sample size	CVDs			Outcome	NOS
				CVDs patients	Severe CVDs/severe cases	Non-severe CVDs/Non-severe cases		
Cen *et al*. [10]	Wuhan, China	Retrospective, multi-centre observational	1007	65	34/287	31/720	Disease progression	7
Chen *et al*. [11]	Taizhou, Zhejiang, China	Retrospective, single-centre, observational	145	1	1/43	0	Disease severity	6
Chen *et al*. [12]	Wuhan, China	Retrospective, single-centre, observational	274	24	17/113	7/161	Death	7
Cocconcelli *et al*. [13]	Padova, Italy	Retrospective, single-centre, observational	102	60	25/31	35/71	Medical care intensity	7
Goyal *et al*. [14]	New York, USA	Retrospective, multi-centre observational	393	54	25/130	29/263	Invasive ventilation	5
Guan *et al*. [15]	Nationwide, China	Retrospective, multi-centre observational	1099	27	10/173	17/926	Disease severity	6
Huang *et al*. [16]	Wuhan, China	Retrospective, single-centre, observational	41	6	3/13	3/28	ICU admission	7
Huang *et al*. [17]	Jiangsu, China	Retrospective, multi-centre, observational	202	5	1/23	4/179	Disease severity	7
Khalil *et al*. [18]	London, England	Retrospective, single-centre, observational	204	24	10/58	14/162	Death	7
Li *et al*. [19]	Wuhan, China	Retrospective, single-centre, observational	544	34	28/269	6/279	Disease severity	7
Ishii *et al*. [20]	Nationwide, Japan	Retrospective, multi-centre observational	345	23	6/23	17/322	Death	7
Shu *et al*. [21]	Wuhan, China	Retrospective, single-centre, observational	571	12	3/26	9/545	Disease progression	7
Wan *et al*. [22]	Chongqing, China	Retrospective, single-centre, observational	135	7	6/40	1/95	Disease severity	7
Wang *et al*. [23]	Fuyang, Anhui, China	Retrospective, single-centre, observational	125	18	NA	NA	Disease severity	6
Wang *et al*. [24]	Wuhan, China	Retrospective, single-centre, observational	110	NA	NA	NA	Disease severity	7
Wang *et al*. [25]	Wuhan, China	Retrospective, single-centre, observational	59	13	10/41	3/18	Death	7
Xie *et al*. [26]	Nationwide, China	Retrospective, multi-centre observational	733	108	64/394	44/339	Death	7
Yang *et al*. [27]	Yichang, China	Retrospective, single-centre, observational	200	11	1/29	10/171	ICU admission	7
Zhan *et al*. [28]	Wuhan, China	Retrospective, single-centre, observational	405	156	77/148	79/257	Disease severity	7
Zhang *et al*. [29]	Wuhan, China	Retrospective, single-centre, observational	140	12	8/58	4/82	Disease severity	7
Zhou *et al*. [30]	Wuhan, China	Retrospective, multi-centre observational	191	15	13/54	2/137	Death	7

### Smoking and COVID-19

#### Smoking history and COVID-19 disease progression

**Fig. 2.**
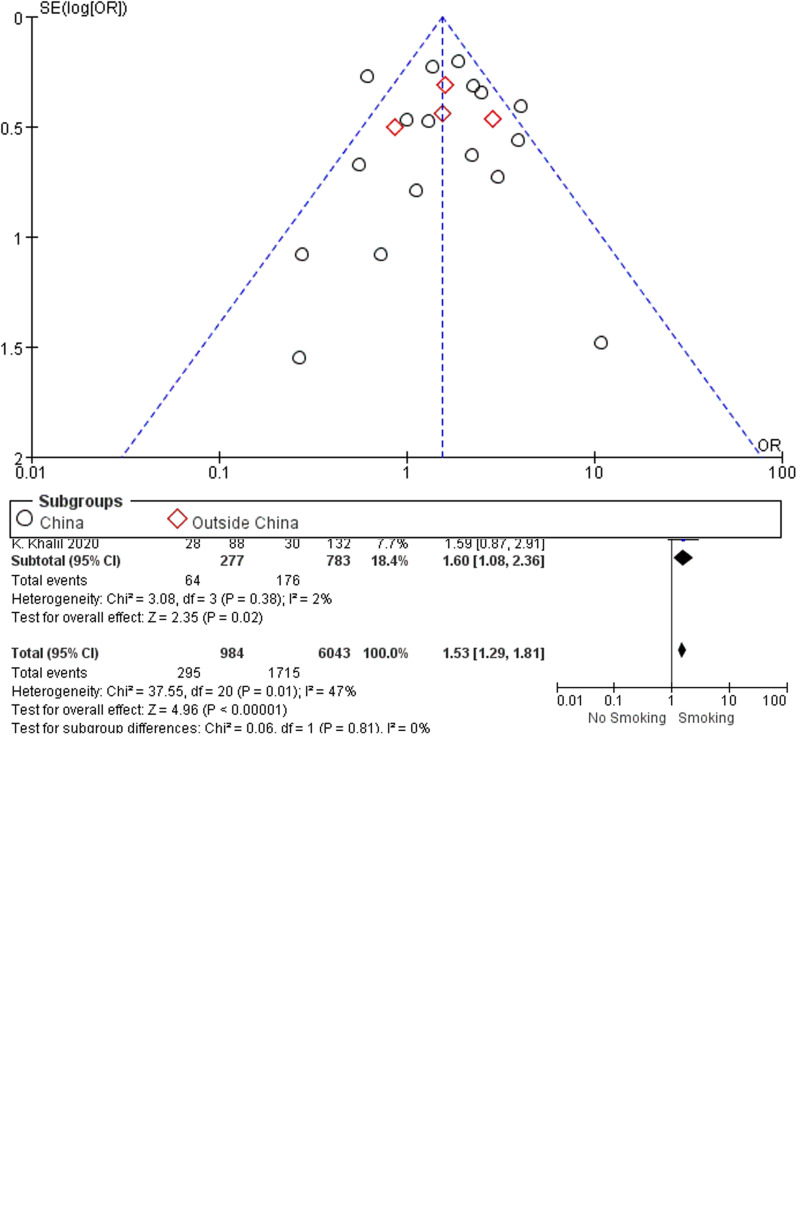
Association between smoking history and COVID-19 disease progression.

A total of 295 (30.0%) of the 984 COVID-19 cases with a smoking history (currently smoking or ex-smoking) experienced disease progression, compared with 28.4% (1715/6043) of the cases without a smoking history. As revealed from the heterogeneity test results, there was mild heterogeneity among the studies (*I*^2^ = 47%, *P* = 0.010), and the fixed-effect model was adopted for meta-analysis. According to the results, (Fig. 2) cases with a smoking history achieved a higher rate of disease progression than cases without a smoking history (OR 1.53, 95% CI 1.29–1.81, *P* < 0.00001). Besides, no difference (*P* = 0.81) was observed between studies from China (17 studies; OR 1.52, 95% CI 1.26–1.83; *I*^2^ = 54%; *P* < 0.00001) and studies outside China (4 studies; OR 1.60, 95% CI 1.08–2.36; *I*^2^ = 2%; *P* = 0.02). Funnel plots were applied to detect publication bias, and the results showed that the funnels were symmetrical and publication bias was less likely.

#### Smoking status and COVID-19 disease progression

**Fig. 3.**
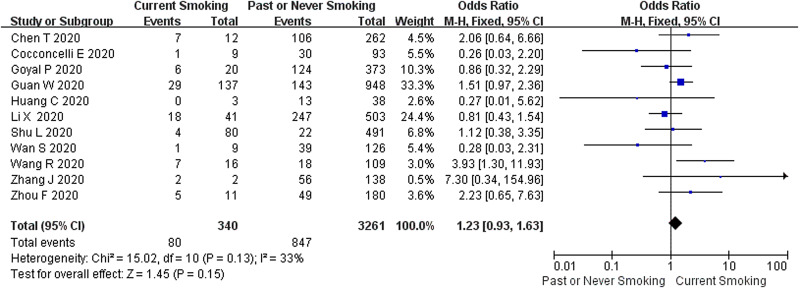
Association between smoking status and COVID-19 disease progression.

Current smoking status was reported in 11 studies comprising 3601 COVID-19 cases, of which 340 (9.4%) were current smokers, resulting in a disease progression rate of 80/340 (23.5%) and 847/3261 (26.0%) were current non-smokers. The heterogeneity test results showed that there was mild heterogeneity among the studies (*I*^2^ = 33%, *P* = 0.13), and the fixed-effect model was used for meta-analysis. As revealed from the results (Fig. 3), no significant difference was identified between current smoking and COVID-19 disease progression (OR 1.23, 95% CI 0.93–1.63, *P* = 0.15).

#### Smoking history and COVID-19 mortality

**Fig. 4.**
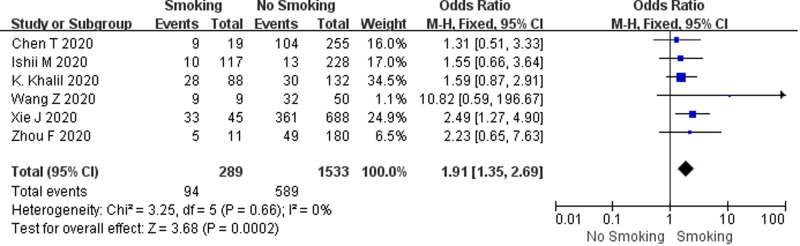
Association between smoking history and COVID-19 mortality.

In this study, a total of six studies, comprising 1822 COVID-19 cases, had an all-cause mortality rate of 37.5% (683/1822) using patient death as the outcome measure. According to the heterogeneity test results, there was no heterogeneity among the six studies on patient death (*I*^2^ = 0%, *P* = 0.66), and the fixed-effect model was employed for meta-analysis. As demonstrated from the results (Fig. 4), cases with a smoking history had a higher mortality rate than cases without a smoking history (OR 1.91, 95% CI 1.35–2.69, *P* = 0.0002).

### Cardiovascular disease and COVID-19

#### Cardiovascular disease and severe COVID-19

**Fig. 5.**
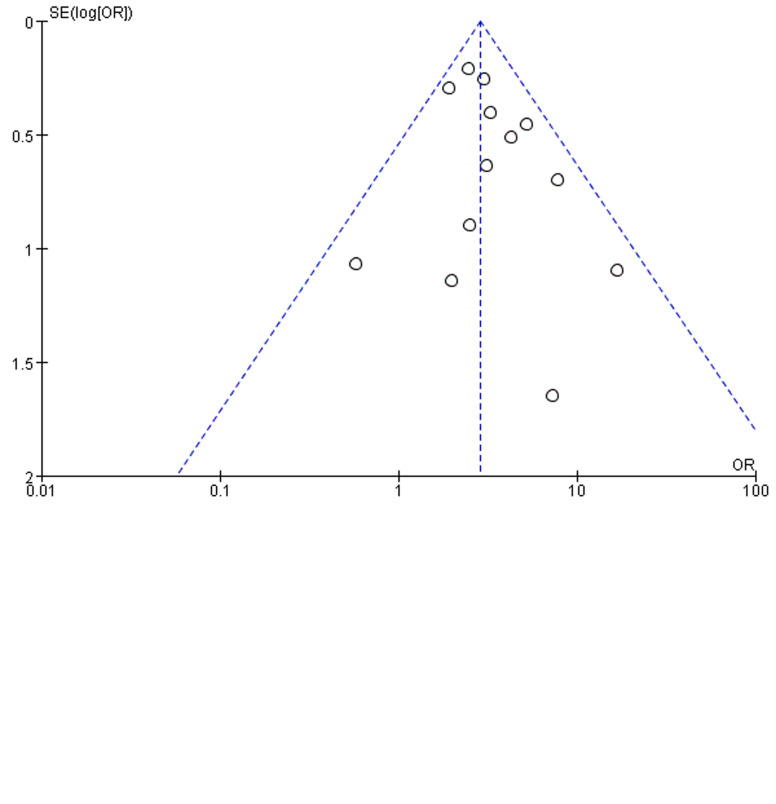
Association between CVD and severe COVID-19.

In this study, a total of 675 cases (9.7%) were diagnosed with COVID-19 and had CVD. A total of 18 studies reported disease results in COVID-19 cases with CVD, 13 of which reported severe disease as an outcome measure. A total of 49.3% (222/450) of COVID-19 cases with CVD developed severe disease in 13 studies, while only 23.1% (1048/4534) of cases without underlying cardiac disease experienced disease progression. According to the heterogeneity test results, there was no heterogeneity among the studies (*I*^2^ = 1%, *P* = 0.44), and the fixed-effect model was employed for meta-analysis. As revealed from the results (Fig. 5), underlying CVD elevated the incidence of severe disease by 2.87 times in COVID-19 cases (OR 2.87, 95% CI 2.29–3.61, *P* < 0.00001). Funnel plots were adopted to detect publication bias, and the results demonstrated that the funnels were symmetrical and publication bias was less likely.

#### Cardiovascular disease and COVID-19 mortality

**Fig. 6.**
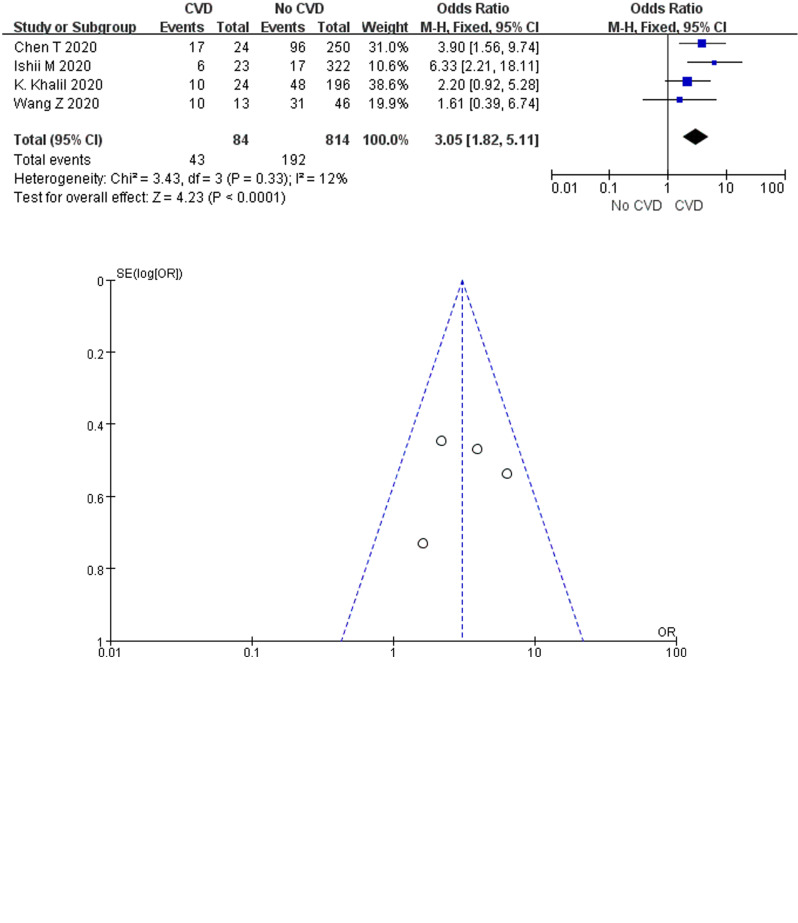
Association between CVD and COVID-19 mortality.

A total of 60.0% (120/207) of COVID-19 cases with CVD died in the six studies, compared with only 34.9% (563/1615) of cases without underlying cardiac disease. According to the heterogeneity test results, significant heterogeneity was identified among the studies (*I*^2^ = 75%, *P* = 0.001), and the random-effects model was employed for meta-analysis. As demonstrated from the results (Fig. 6a), underlying CVD made a large association with mortality in COVID-19 cases (OR 3.26, 95% CI 1.53–6.94, *P* = 0.002).

Two studies of Xie *et al*. [26] and Zhou *et al*. [30] were, respectively, identified as the main sources of heterogeneity by excluding line sensitivity analysis one by one for the six studies included. Analysis of the remaining four studies after excluding the mentioned two confirmed that all-cause mortality was 26.2% (235/898) in COVID-19 cases. The results of heterogeneity test showed that there was no heterogeneity among the four studies (*I*^2^ = 12%, *P* = 0.33), and fixed effect model was used for meta-analysis. The results showed (Fig. 6b) that underlying CVD increased mortality by 3.05-fold in COVID-19 cases (OR 3.05, 95% CI 1.82–5.11, *P* < 0.0001). Funnel plots were applied to detect publication bias, and the results showed that the funnels were symmetrical and publication bias was less likely.

## Discussion

A total of 21 studies were included in this meta-analysis, and the results showed that 14.0% of COVID-19 cases had smoking history, while 4.8% were still smoking. Smoking history displayed a significant association with COVID-19 disease progression (OR 1.53). Moreover, we found that the rate of CVD in COVID-19 cases was 9.7%, and the rate of severe disease (49.3%) and mortality (51.2%) were significantly increased in such cases, suggesting that underlying CVD is significantly associated with poor prognosis in COVID-19 cases.

As suggested by the World Health Organization's 2018 Global Adult Tobacco Report, the adult smoking rate in China continues to maintain a high value of 26.6%, and more than half (50.5%) of men are still smoking [31]. Wang *et al*. collected the data of national health survey in China in 2003, 2008 and 2013, respectively. As reported by the study, the proportion of smokers in China was 26.0%, 24.9% and 25.2%, respectively, demonstrating that the smoking status in China has not been improved over the past decade since the implementation of the Tobacco Control Regulations [32]. However, the smoking rate of COVID-19 cases obtained in this study was only 14.0%, which was much lower than the current international epidemiological survey data and study expectations. It is considered that besides the representativeness of the studies included, and the need for further adjustment of the relevant factors in methodology, factors (e.g. the considerable critically ill cases at the early stage of the epidemic, lack of medical history and records) should be considered. Furthermore, relevant information on smoking habits, number and patient complications in subsequent clinical work or even in response to the next epidemic should be rigorously collected to more effectively guide disease treatment.

Some studies have proposed relevant conclusions and opinions on the association between smoking and COVID-19 disease progression. Karanasos *et al*. [6] found that smoking increased the risk of severe disease in COVID-19 cases, particularly in younger cases without diabetes (OR 1.34, 95% CI 1.07–1.67). Patanavanich *et al*. [33] proposed that smoking displayed a significant association with COVID-19 disease progression (OR 1.91, 95% CI 1.42–2.59). The study by Farsalinos *et al*. [34] indicated that cases who were currently smoking had a worse prognosis than cases without a history of smoking (OR 1.53, 95% CI 1.06 −2.20). The study by Zhao *et al*. [35] also proposed that active and persistent smoking can increase the incidence rate of severe COVID-19 by nearly 2-fold (OR 1.98, 95% CI 1.29–3.05). Lowe *et al*. [36] recently found patients who smoked more than 30 pack-years had a higher odds of hospitalisation (OR 2.25, 95% CI 1.76–2.88), and were more likely to die following a COVID-19 diagnosis (OR 1.89, 95% CI 1.29–2.76) when compared with never smokers. The above studies and analyses support the conclusions drawn from our study, but some studies still draw opposite conclusions. The study by Lippi *et al*. [5] reported that active smoking was not associated with COVID-19 severity (OR 1.69; 95% CI 0.41–6.92; *P* = 0.254). Similar conclusions were drawn by Lombardi *et al*. [37], who concluded that active smoking was not significantly associated with COVID-19 severity. However, Lippi *et al*. did not perform a risk of bias assessment of the studies included, Lombardi *et al*. only investigated in-hospital *vs.* non-in-hospital mortality using active smoking as an exposure factor, and the study was limited to the induction and description of clinical data rather than a systematic review and analysis. Therefore, the conclusions of the mentioned two studies should be objectively analysed and interpreted. Comparison between the six systematic reviews or meta-analyses and the present review among smoking is listed in Table 3.

**Table 3.** Comparison of the six systematic reviews or meta-analyses with the present review among smoking

	Title	Country	*N* studies & type; country	Time period searched	Result	Conclusion
Vardavas *et al*. [3]	COVID-19 and smoking: A systematic review of the evidence	USA	5 retrospective studies; China 5	1 January 2019 to 17 March 2020	The smokers were 1.4 times more likely (95% CI 0.98–2.00) to have severe symptoms of COVID-19 and approximately 2.4 times more likely to be admitted to an ICU, need mechanical ventilation or die compared to non-smokers (95% CI 1.43–4.04)	Smoking is most likely associated with the negative progression and adverse outcomes of COVID-19
Lippi *et al*. [5]	Active smoking is not associated with severity of coronavirus disease 2019 (COVID-19)	Italy	5 retrospective studies; China 5	1 January 2019 to 9 March 2020	No significant association could be found between active smoking and severity of COVID-19 (OR 1.69, 95% CI 0.41–6.92, *P* = 0.254).	Active smoking does not apparently seem to be significantly associated with enhanced risk of progressing towards severe disease in COVID-19
Karanasos *et al*. [6]	Impact of Smoking Status on Disease Severity and Mortality of Hospitalised Patients with COVID-19 Infection: A Systematic Review and Meta-analysis	Greece	22 retrospective studies; China 20 USA 2	1 September 2019 to 4 May 2020	Smoking modestly increased the risk for the combined end point of disease severity (OR 1.34, 95% CI 1.07–1.67, *I*^2^ = 45%); In studies with low (<15%) prevalence of diabetes, smoking increased the risk for severe disease (OR 1.66, 95% CI 1.26–2.18, *I*^2^ = 34%)	Data suggest a possible adverse impact of smoking on disease severity and mortality of hospitalised COVID-19 patients, which is more pronounced in younger patients without diabetes
Patanavanich *et al*. [33]	Smoking Is Associated With COVID-19 Progression: A Meta-analysis	USA	19 retrospective studies; China 16, Korea 1, USA 2	1 January 2020 to 28 April 2020	The meta-analysis showed a significant association between smoking and progression of COVID-19 (OR 1.91, 95% CI 1.42–2.59, *P* = 0.001)	Smoking is a risk factor for progression of COVID-19, with smokers having higher odds of COVID-19 progression than never smokers
Farsalinos *et al*. [34]	Current smoking, former smoking, and adverse outcome among hospitalised COVID-19 patients: a systematic review and meta-analysis	Greece	18 retrospective studies; China 15, Korea 1, USA 2	Until 25 April 2020	Current smokers were more likely to have an adverse outcome compared with non-current smokers (OR 1.53, 95% CI 1.06–2.20, *P* = 0.022) but less likely compared with former smokers (OR 0.42, 95% CI 0.27–0.74, *P* = 0.003)	Hospitalised current smokers had higher odds compared with non-current smokers but lower odds compared with former smokers for an adverse outcome. Smoking cannot be considered a protective measure for COVID-19
Zhao *et al*. [35]	The impact of COPD and smoking history on the severity of COVID-19: A systemic review and meta-analysis	China	11 retrospective studies; China 11	December 2019 to 22 March 2020	The pooled OR of COPD and the development of severe COVID-19 was 4.38 (95% CI 2.34–8.20), while the OR of ongoing smoking was 1.98 (95% CI 1.29–3.05)	COPD and ongoing smoking history attribute to the worse progression and outcome of COVID-19
Present review	Association of smoking and CVD with disease progression in COVID-19: A systematic review and meta-analysis	China	21 retrospective studies; China 17, USA 1, UK 1, Japan 1, Italy 1	1 January 2020 to 6 October 2020	Cases with a history of smoking achieved a higher rate of COVID-19 disease progression as opposed to those having not smoked (OR 1.53, 95% CI 1.29–1.81, *P* < 0.00001), while no significant association could be found between smoking status and COVID-19 disease progression (OR 1.23, 95% CI 0.93–1.63, *P* = 0.15). Besides, smoking history elevated the mortality rate by 1.91-fold (OR 1.91, 95% CI 1.35–2.69, *P* = 0.0002)	Smoking displays a strong association with COVID-19 disease progression and mortality, and intensive tobacco control is imperative

Recent studies suggest that active smokers are underrepresented among patients with COVID-19 [38]. ‘Smoker's paradox’ has been claimed recently and a false impression that smoking can give protection against COVID-19 was perceived by the general population. Changeux *et al*. [39] have proposed the nicotinic hypothesis. Nicotinic acetylcholine receptor (nAChR) was found to play a role in the COVID-19 inflammatory syndrome. Due to this finding, the hypothesis suggests that nicotine could reduce SARS-CoV-2 infection and alleviate COVID-19 progression by competing with SARS-CoV-2 in binding to the nAChR. Besides, the anti-inflammatory effect of nicotine and the inhibiting effect of nitrogen monoxide in SARS-CoV-2 replication might also support the hypothesis [40]. But the hypothesis could only be explained by exposure to nicotine, rather than the cigarette smoke with thousands of harmful chemicals. Since only questionable data were reported, and evidence is inadequate, the protective effects of nicotine should not be inferred determinedly. However, nicotinic hypothesis offers the public an alternative vision and a potential treatment of COVID-19. Placebo-controlled trials should be performed to assess the viability of nicotine as a therapeutic option.

Although the major symptoms of COVID-19 do not arise from the cardiovascular system, data analysis shows that more COVID-19 cases have CVD, probably related to the older age of COVID-19 cases and their male predominance [16]. The present study indicated that the rate of coexisting CVD in COVID-19 cases was 9.7%, supporting this view as well. Cases subject to underlying CVDs were found to be at higher risk for acute cardiovascular events, thromboembolism, infection and disease progression after SARS-COV-2 infection [41]. In the study, the proportion of severe disease and death in such cases was found to be significantly elevated as well. Likewise, considerable existing studies on respiratory infectious diseases (e.g., SARS and MERS) have also reported related cardiovascular complications, the basic pathophysiological mechanism of which is that viral infection increases systemic inflammatory response, thereby causing an imbalance in cardiac metabolic supply and demand [42]. For this reason, the special situation of cardiovascular system diseases combined with COVID-19 refers to a major focus and difficulty in this outbreak treatment.

A study of 138 SARS-COV-2 infected cases found that 7.2% had myocardial injury, while the proportion of myocardial injury in SARS-COV-2 infected cases admitted to ICU was 22.2%, which was significantly higher than that in SARS-COV-2 infected cases admitted to general ward (2.0%) [8]. Acute myocardial injury (77%) and heart failure (49%) were common complications and associated with higher mortality in COVID-19 cases regardless of whether the cases had a relevant previous medical history [12]. As highlighted by another study, myocardial injury displayed a significant association with mortality in COVID-19, while elevated TnT levels had some cautionary role [43].

The specific mechanism by which SARS-COV-2 causes cardiopulmonary injury is unknown. Studies have shown that SARS-COV-2 enters cells largely through spike protein on the viral surface and ACE2 in host respiratory epithelial cells after entering the human body, causing down-regulation of ACE2 and increased angiotensin II (Ang II) levels in the body; as a result, multiple organ dysfunction is induced (e.g. cardiovascular lesions and lung injury) [44]. It has been confirmed in the existing literature that Ang II levels are significantly elevated in cases infected with SARS-COV-2, and the degree of its increase is associated with the severity of the disease, suggesting that SARS-COV-2 may activate the renin−angiotensin system (RAS) to induce an inflammatory storm and cause poor prognosis of cases [44].

It is necessary to pay attention to the treatment of underlying CVDs in cases with COVID-19, as well as to guard against the side effects of drugs and avoid aggravating the condition. Existing studies suggest that the use of ACEI and ARB drugs may cause potential harm, and their application has sparked numerous controversies since they may cause ACE2 to up-regulated and accelerate SARS-COV-2 infection and lung injury [45]. However, large-sample clinical studies on the effects of ACEIs or ARBs on lung injury in infected cases have been rarely conducted [46]. At present, there is no clinical or scientific evidence to support the change or discontinuation of ACEI/ARB drugs in cases with COVID-19. In addition, antiviral drugs (e.g., lopinavir/ritonavir) although used as first-line anti-SARS-COV-2 drugs, have common cardiovascular adverse effects including hypertension, prolonged P-R interval and torsades de pointes [47]. Chloroquine phosphate is capable of inducing serious cardiovascular adverse effects (e.g. arrhythmias, shock and Asperger's syndrome) [48]. The adverse reactions of antiviral drugs and drug−drug interactions still need to be further observed in clinical practice.

Smoking can be harmful to all the organs in the human body. It is a common risk factor for CVD, cancer, diabetes and chronic respiratory disease. Cardiovascular morbidity and mortality attributed to smoking remains a global health problem, especially in low-income countries that lack smoking restriction policies. As revealed from the existing studies, CVD risk in light smokers decreases to baseline levels within 5 years after smoking cessation, while CVD risk in heavy smokers does not decrease to baseline levels in never smokers until 15 years after smoking cessation [49]. The mechanism of smoking leading to the occurrence and development of coronary heart disease may consist of the following processes: damaging endothelial function and inducing vasospasm, aggravating inflammatory response, disrupting the balance of coagulation and fibrinolysis system in the body, as well as promoting abnormal lipid metabolism. With the flooding of tobacco and long-term, extensive and massive smoking by the people, the age of onset of CVD is gradually younger, the prevalence tends to rise, and the cardiovascular basis and cardiac functional reserve are decreasing. It was also demonstrated that ACE2 expression was up-regulated in airway epithelial cells of smokers, causing smokers to be more susceptible to COVID-19 [50]. After the outbreak of COVID-19, severe infections and inflammatory factor storms attributed to SARS-COV-2 caused a second blow to the already fragile heart of cases with CVD and progressed. In addition, the relative tension of medical resources at the early stage of the epidemic, the closure of considerable pharmacies and the closed management of residential areas make some cases with stable CVD experience drug interruption and cannot complete the self-management of the disease. Cases subject to CVD can be suggested to have a higher risk of conversion to severe and critical illness and even life-threatening during the COVID-19 epidemic, while smoking impacts the development of CVD and COVID-19.

In brief, COVID-19 has plunged both China and the world into a difficult period in history. The long-term impact of this outbreak may profoundly change the world. With the society gradually recovering from the epidemic, the anti-smoking campaign should continue to persist and facilitate the people's health and sustainable economic and social development.

Several limitations are revealed here. First, the studies included in this paper were retrospective cohort studies, and most of the studies were from China. We cannot exclude the possibility of ethnic differences in smoking and susceptibility to severe COVID-19. Yet, the use of aggregate data may preclude adjustments for certain confounders such as age, gender and comorbidities reported to be predictive of disease severity. Further prospective cohort studies dedicated to analysing this matter should take into consideration of such adjustments. Second, some studies did not clearly distinguish smoking history from current smoking status. Third, the association between duration or cumulative smoking exposure and COVID-19 severity could not be assessed in our study since no relevant data were reported in the included studies. Despite all the pressure in the pandemic, Smoking Index should be promoted in clinical settings, and detailed data about smoking history needs to be collected by healthcare professionals/researchers adequately. Finally, most studies separate CVD population from hypertension population, resulting in overlapping and missing data.

Despite the mentioned limitations, this meta-analysis assessed the association between smoking, CVD and disease progression in COVID-19. There were many included literatures with large sample size and no significant publication bias. Thus, the reliability of meta-analysis results remains strong.

## Conclusion

It is currently evidenced that smoking displays a strong association with COVID-19 disease progression and mortality. It is imperative to curb tobacco flooding. Moreover, cases with CVD have a significantly elevated risk of poor disease progression and death when subject to COVID-19. The association between COVID-19 and CVD, and the potential effect exerted by smoking in the development of the two, require in-depth verifications by larger and higher quality studies.

## Data Availability

The data that support the findings of this study are available from the corresponding author upon reasonable request.

## Supplementary material

For supplementary material accompanying this paper visit http://dx.doi.org/10.1017/S0950268821001138. click here to view supplementary material

## References

[1] Lancet T (2020) Emerging understandings of 2019-nCoV. Lancet (London, England) 395, 311. 10.1016/S0140-6736(20)30186-0 PMC7134625 31986259

[2] WHO (2021) Coronavirus disease 2019 (COVID-19) Weekly epidemiological update – 27 March 2021 [EB/OL]. [2021-03-27].

[3] Vardavas CI (2020) COVID-19 and smoking: a systematic review of the evidence. Tobacco Induced Diseases 18, 20. 32206052 10.18332/tid/119324 PMC7083240

[4] Han L (2019) Smoking and influenza-associated morbidity and mortality: a systematic review and meta-analysis. Epidemiology (Cambridge, Mass.) 30, 405–417. 10.1097/EDE.0000000000000984 30789425

[5] Lippi G (2020) Active smoking is not associated with severity of coronavirus disease 2019 (COVID-19). European Journal of Internal Medicine 75, 107–108. 32192856 10.1016/j.ejim.2020.03.014 PMC7118593

[6] Karanasos A (2020) Impact of smoking status on disease severity and mortality of hospitalized patients with COVID-19 infection: a systematic review and meta-analysis. Nicotine & Tobacco Research: Official Journal of the Society for Research on Nicotine and Tobacco 22, 1657–1659. 32564072 10.1093/ntr/ntaa107 PMC7337737

[7] Zhu N (2020) A novel coronavirus from patients with pneumonia in China, 2019. The New England Journal of Medicine 382, 727–733. 31978945 10.1056/NEJMoa2001017 PMC7092803

[8] Wang D (2020) Clinical characteristics of 138 hospitalized patients with 2019 novel coronavirus-infected pneumonia in Wuhan, China. JAMA 323, 1061–1069. 32031570 10.1001/jama.2020.1585 PMC7042881

[9] Hackshaw A (2018) Low cigarette consumption and risk of coronary heart disease and stroke: meta-analysis of 141 cohort studies in 55 study reports. BMJ: British Medical Journal 360, j5855. 29367388 10.1136/bmj.j5855 PMC5781309

[10] Cen Y (2020) Risk factors for disease progression in patients with mild to moderate coronavirus disease 2019 − a multi-centre observational study. Clinical Microbiology and Infection: the Official Publication of the European Society of Clinical Microbiology and Infectious Diseases 26, 1242–1247. 10.1016/j.cmi.2020.05.041 PMC7280135 32526275

[11] Chen Q (2020) Clinical characteristics of 145 patients with corona virus disease 2019 (COVID-19) in Taizhou, Zhejiang, China. Infection 48, 543–551. 32342479 10.1007/s15010-020-01432-5 PMC7186187

[12] Chen T (2020) Clinical characteristics of 113 deceased patients with coronavirus disease 2019: retrospective study. BMJ: British Medical Journal 368, m1091. 32217556 10.1136/bmj.m1091 PMC7190011

[13] Cocconcelli E (2020) Clinical features and chest imaging as predictors of intensity of care in patients with COVID-19. Journal of Clinical Medicine 9(9), 2990. 10.3390/jcm9092990 PMC7565657 32947904

[14] Goyal P (2020) Clinical characteristics of COVID-19 in New York City. The New England Journal of Medicine 382, 2372–2374. 32302078 10.1056/NEJMc2010419 PMC7182018

[15] Guan WJ (2020) Clinical characteristics of coronavirus disease 2019 in China. The New England Journal of Medicine 382, 1708–1720. 32109013 10.1056/NEJMoa2002032 PMC7092819

[16] Huang C (2020) Clinical features of patients infected with 2019 novel coronavirus in Wuhan, China. Lancet (London, England) 395, 497–506. 10.1016/S0140-6736(20)30183-5 PMC7159299 31986264

[17] Huang R (2020) Clinical findings of patients with coronavirus disease 2019 in Jiangsu province, China: a retrospective, multi-center study. PLoS Neglected Tropical Diseases 14, e0008280. 32384078 10.1371/journal.pntd.0008280 PMC7239492

[18] Khalil K (2020) Clinical characteristics and 28-day mortality of medical patients admitted with COVID-19 to a central London teaching hospital. The Journal of Infection 81, e85–e89. 10.1016/j.jinf.2020.06.027 PMC7299867 32562795

[19] Li X (2020) Risk factors for severity and mortality in adult COVID-19 inpatients in Wuhan. The Journal of Allergy and Clinical Immunology 146, 110–118. 32294485 10.1016/j.jaci.2020.04.006 PMC7152876

[20] Ishii M (2020) Clinical characteristics of 345 patients with coronavirus disease 2019 in Japan: a multicenter retrospective study. The Journal of Infection 81, e3–e5. 32920063 10.1016/j.jinf.2020.08.052 PMC7482596

[21] Shu L (2020) Clinical characteristics of moderate COVID-19 patients aggravation in Wuhan Stadium Cabin Hospital: a 571 cases of retrospective cohort study. Journal of Medical Virology 93, 1133–1140. 32779760 10.1002/jmv.26414 PMC7436609

[22] Wan S (2020) Clinical features and treatment of COVID-19 patients in northeast Chongqing. Journal of Medical Virology 92, 797–806. 32198776 10.1002/jmv.25783 PMC7228368

[23] Wang R (2020) Epidemiological and clinical features of 125 hospitalized patients with COVID-19 in Fuyang, Anhui, China. International Journal of Infectious Diseases: IJID: Official Publication of the International Society for Infectious Diseases 95, 421–428. 32289565 10.1016/j.ijid.2020.03.070 PMC7151431

[24] Wang Y (2020) Clinical characteristics of patients with severe pneumonia caused by the SARS-CoV-2 in Wuhan, China. Respiration; International Review of Thoracic Diseases 99, 649–657. 32841948 10.1159/000507940 PMC7490495

[25] Wang ZH (2020) Critically ill patients with coronavirus disease 2019 in a designated ICU: clinical features and predictors for mortality. Risk Management and Healthcare Policy 13, 833–845. 32765138 10.2147/RMHP.S263095 PMC7381092

[26] Xie J (2020) Clinical characteristics and outcomes of critically ill patients with novel coronavirus infectious disease (COVID-19) in China: a retrospective multicenter study. Intensive Care Medicine 46, 1863–1872. 32816098 10.1007/s00134-020-06211-2 PMC7439240

[27] Yang L (2020) Epidemiological and clinical features of 200 hospitalized patients with corona virus disease 2019 outside Wuhan, China: a descriptive study. Journal of Clinical Virology: The Official Publication of the Pan American Society for Clinical Virology 129, 104475. 32485619 10.1016/j.jcv.2020.104475 PMC7250074

[28] Zhan T (2020) Retrospective analysis of clinical characteristics of 405 patients with COVID-19. The Journal of International Medical Research 48, 300060520949039. 10.1177/0300060520949039 PMC7459177 32865077

[29] Zhang JJ (2020) Clinical characteristics of 140 patients infected with SARS-CoV-2 in Wuhan, China. Allergy 75, 1730–1741. 32077115 10.1111/all.14238

[30] Zhou F (2020) Clinical course and risk factors for mortality of adult inpatients with COVID-19 in Wuhan, China: a retrospective cohort study. Lancet (London, England) 395, 1054–1062. 10.1016/S0140-6736(20)30566-3 PMC7270627 32171076

[31] Chinese Center for Disease Control and Prevention (2018) Global Adult Tobacco Survey (GATS). Fact sheet China 2018 [EB/OL]. [2020-12-18].

[32] Wang M (2019) Trends in smoking prevalence and implication for chronic diseases in China: serial national cross-sectional surveys from 2003 to 2013. The Lancet. Respiratory Medicine 7, 35–45. 30482646 10.1016/S2213-2600(18)30432-6

[33] Patanavanich R (2020) Smoking is associated with COVID-19 progression: a meta-analysis. Nicotine & Tobacco Research: Official Journal of the Society for Research on Nicotine and Tobacco 22, 1653–1656. 32399563 10.1093/ntr/ntaa082 PMC7239135

[34] Farsalinos K (2020) Current smoking, former smoking, and adverse outcome among hospitalized COVID-19 patients: a systematic review and meta-analysis. Therapeutic Advances in Chronic Disease 11, 2040622320935765. 10.1177/2040622320935765 PMC7318805 32637059

[35] Zhao Q (2020) The impact of COPD and smoking history on the severity of COVID-19: a systemic review and meta-analysis. Journal of Medical Virology 92, 1915–1921. 32293753 10.1002/jmv.25889 PMC7262275

[36] Lowe KE (2021) Association of smoking and cumulative pack-year exposure with COVID-19 outcomes in the Cleveland clinic COVID-19 registry. JAMA Internal Medicine 181, 709–711. 33492361 10.1001/jamainternmed.2020.8360 PMC7835916

[37] Lombardi C (2020) Smoking and COVID-19, the paradox to discover: an Italian retrospective, observational study in hospitalized and non-hospitalized patients. Medical Hypotheses 146, 110391. 33261917 10.1016/j.mehy.2020.110391 PMC7659913

[38] Tsigaris P (2020) Smoking prevalence and COVID-19 in Europe. Nicotine & Tobacco Research: Official Journal of the Society for Research on Nicotine and Tobacco 22, 1646–1649. 32609839 10.1093/ntr/ntaa121 PMC7337760

[39] Changeux JP (2020) A nicotinic hypothesis for COVID-19 with preventive and therapeutic implications. Comptes Rendus Biologies 343, 33–39. 32720486 10.5802/crbiol.8

[40] Usman MS (2020) Is there a smoker's paradox in COVID-19. BMJ Evidence-Based Medicine, bmjebm-2020-111492. 10.1136/bmjebm-2020-111492 32788164

[41] Zheng YY (2020) COVID-19 and the cardiovascular system. Nature Reviews. Cardiology 17, 259–260. 10.1038/s41569-020-0360-5 PMC7095524 32139904

[42] Alhogbani T (2016) Acute myocarditis associated with novel Middle East respiratory syndrome coronavirus. Annals of Saudi Medicine 36, 78–80. 26922692 10.5144/0256-4947.2016.78 PMC6074274

[43] Guo T (2020) Cardiovascular implications of fatal outcomes of patients with coronavirus disease 2019 (COVID-19). JAMA Cardiology 5, 811–818. 32219356 10.1001/jamacardio.2020.1017 PMC7101506

[44] Liu Y (2020) Clinical and biochemical indexes from 2019-nCoV infected patients linked to viral loads and lung injury. Science China. Life Sciences 63, 364–374. 32048163 10.1007/s11427-020-1643-8 PMC7088566

[45] Callera GE (2016) Differential renal effects of candesartan at high and ultra-high doses in diabetic mice-potential role of the ACE2/AT2R/Mas axis. Bioscience Reports 36(5), e00398. 27612496 10.1042/BSR20160344 PMC5091470

[46] Vaduganathan M (2020) Renin-angiotensin-aldosterone system inhibitors in patients with COVID-19. The New England Journal of Medicine 382, 1653–1659. 32227760 10.1056/NEJMsr2005760 PMC7121452

[47] Reyskens KM (2013) Cardio-metabolic effects of HIV protease inhibitors (lopinavir/ritonavir). PLoS One 8, e73347. 24098634 10.1371/journal.pone.0073347 PMC3787040

[48] Pareek A (2018) Metabolic and cardiovascular benefits of hydroxychloroquine: exploration in a wider population at high CV risk. Annals of the Rheumatic Diseases 77, e59. 29030360 10.1136/annrheumdis-2017-212499

[49] Duncan MS (2019) Association of smoking cessation with subsequent risk of cardiovascular disease. JAMA 322, 642–650. 31429895 10.1001/jama.2019.10298 PMC6704757

[50] Leung JM (2020) ACE-2 expression in the small airway epithelia of smokers and COPD patients: implications for COVID-19. The European Respiratory Journal 55(5), 2000688. 32269089 10.1183/13993003.00688-2020 PMC7144263

