# Supplementary material for: Association of smoking and cardiovascular disease with disease progression in COVID-19: a systematic review and meta-analysis
Source: Epidemiol Infect. 2021 May 12;149:e143. doi: 10.1017/S0950268821001138 (PMC8220032; doi:10.1017/S0950268821001138)
Supplement: Supplementary file 1 [file S0950268821001138sup001.docx]

**Association of smoking and cardiovascular disease with disease progression in COVID-19: A systematic review and meta-analysis**

Shiwei Kang , Xiaowei Gong , Yadong Yuan*

**Supplementary Methods**

**Pubmed search strategy**

#1 COVID-19 [Supplementary Concept]

#2 2019 novel coronavirus disease[Title/Abstract]

#3 COVID-19 pandemic [Title/Abstract]

#4 SARS-CoV-2 infection [Title/Abstract]

#5 COVID-19 virus disease [Title/Abstract]

#6 2019 novel coronavirus infection [Title/Abstract]

#7 SARS-COV-2 infection [Title/Abstract]

#8 coronavirus disease 2019 [Title/Abstract]

#9 coronavirus disease-19 [Title/Abstract]

#10 SARS-COV-2 disease [Title/Abstract]

#11 COVID-19 virus infection [Title/Abstract]

#12 #1 OR #2 OR #3 OR #4 OR #5 OR #6 OR #7 OR #8 OR #9 OR #10 OR #11

#13 clinical characteristics [Title/Abstract]

#14 clinical features [Title/Abstract]

#15 smoking [MeSH]

#16 smokers [MeSH]

#17 tobacco [MeSH]

#18 cigarette smoking [MeSH]

#19 nicotine [MeSH]

#20 #13 OR #14 OR #15 OR #16 OR #17 OR #18 OR #19

#21 #12 AND #20
